# The effects of photodynamic therapy on leukemia cells mediated by KillerRed, a genetically encoded fluorescent protein photosensitizer

**DOI:** 10.1186/s12885-019-6124-0

**Published:** 2019-10-07

**Authors:** Meng Yuan, Chengcheng Liu, Jiao Li, Wenpeng Ma, Xiaozhuo Yu, Ping Zhang, Yanhong Ji

**Affiliations:** Department of Pathogenic Microbiology & Immunology, School of Basic Medical Sciences, Xi’an Jiaotong University Health Science Center, 76 West Yanta Road, Xi’an, 710061 People’s Republic of China

**Keywords:** Leukemia, KillerRed, Photodynamic therapy, Cell proliferation, Apoptosis

## Abstract

**Background:**

Leukemia is a cancer of blood and bone marrow cells, causing about 300,000 deaths worldwide. Photodynamic therapy (PDT) is a promising alternative for the treatment of malignant tumors. KillerRed is a genetically encoded red fluorescent protein photosensitizer (PS). In this study, we aimed to investigate the effects of KillerRed-mediated PDT on chronic myelogenous leukemia K562 cells, acute monocytic leukemia NB4 cells, and acute monocytic leukemia THP1 cells.

**Methods:**

KillerRed was expressed in *Escherichia coli* cells, purified by Q-Sepharose column, and confirmed by western-blotting. The PDT effect on cell proliferation was evaluated by Cell Counting Kit-8 (CCK-8). Cell apoptosis was determined by PE Annexin V/7-AAD staining and flow cytometry. The distribution of KillerRed in leukemia cells was detected by confocal laser scanning microscopy (CLSM) and western-blotting. The ROS generation was measured by flow cytometry.

**Results:**

Pure KillerRed was obtained with a yield of about 37 mg per liter of bacterial cells. KillerRed photodynamic inactivated the leukemia cells in a concentration-dependent manner, but exhibited no obvious dark toxicity. PDT mediated by KillerRed could also induce apoptotic response (mainly early apoptosis) in the three cell lines. The CLSM imaging indicated that KillerRed was distributed within the cytoplasm and nuclei of leukemia cells, causing damages to the cytoplasm and leaving the nuclear envelope intact during light irradiation. KillerRed distributed both in the cytosol and nuclei was confirmed by western blotting, and ROS significantly increased in PDT treated cells compared to the cells treated with KillerRed alone.

**Conclusions:**

Our studies demonstrated that KillerRed-mediated PDT could effectively inactivate K562, NB4, and THP1 leukemia cells and trigger cell apoptosis, and it has potential to be used individually or complementally, in the treatment of leukemia.

## Background

Leukemia, defined as cancer of the blood and bone marrow cells, usually results in high numbers of abnormally immature white blood cells which distribute at the early steps of the hematopoietic hierarchy [1], and cause a lot of symptoms such as bleeding, fever, feeling tired, and an increased risk of infections. Based on the primary type of cell affected, leukemia can be categorized into lymphocytic leukemia, which occurs in the common lymphoid progenitor lineage, and myeloid leukemia, which develops from the common myeloid progenitor lineage. Based on the disease course, leukemia can also be classified into acute and chronic leukemia. Acute leukemia is usually characterized by overgrowth and rapid accumulation of immature malignant blood cells. However, it is also worth noting that acute leukemia can present with a pancytopaenia, since the disorder arises in the bone marrow and the excess blasts crowd out normal haematopoiesis. Chronic leukemia often exhibits a slower overgrowth of mature blood cells, and may take months to years to progress [2]. According to the status report on the global burden of cancer using the GLOBOCAN 2018 estimates produced by the International Agency for Research on Cancer (IARC), there will be an estimated 437,033 new leukemia cases, causing 309,006 deaths in 2018 worldwide [3]. In China, the data from the National Central Cancer Registry of China (NCCR) demonstrates that the incidence and mortality of leukemia are estimated 75,300 and 53,400, respectively, in 2015 [4]. Although the current treatment for leukemia mainly involves allogenous stem cell transplantation, radiation therapy, and chemotherapy, these therapies could lead to serious late effects such as higher risk of infections, graft-versus-host disease (GVHD), cytotoxicity to normal cells, and especially drug-resistance [5]. Therefore, much more attention has been focused on searching for alternative approaches.

Photodynamic therapy (PDT) is a promising alternative to radiation therapy or chemotherapy for the treatment of malignant tumors [6]. It utilizes near-infrared or visible light of the appropriate wavelength to excite a photosensitizer (PS) from a ground to a triplet state. The triplet state PS reacts with molecule oxygen present in and around the tumor cells to generate singlet oxygen (^1^O_2_) or other reactive oxygen species (ROS). These ROS molecules are able to destroy tumors by multifactorial mechanisms, including directly inducing death of cancer cells by necrosis and/or apoptosis [7], destruction of tumor vasculature as an anti-angiogenesis effect [8], and also the stimulation of the host immune system to recognize, track down and destroy any remaining tumor cells [9]. With respect to radiation therapy and chemotherapy which are mostly immunosuppressive, PDT shows much lower toxic side effects because PS accumulates in significantly higher concentrations in cancer cells than in normal cells [10]; and PDT can be locally applied onto a specific region by selectively illuminating the lesion, while leaving normal tissues untouched [11]. Another advantage may be that PDT does not lead to cumulative toxicity in the patient, and there is no known maximum cumulative dose as exists with both radiation therapy and chemotherapy [12]. Moreover, it is believed that the unique mechanisms of PDT producing damages on tumor cells and microenvironment could be utilized to overcome cancer drug-resistance, to mitigate the compensatory induction of survival pathways, and even to re-sensitize drug-resistant cells to chemotherapy [13]. For these reasons, in recent years, PDT has become the main subject of intense investigation as a possible treatment modality for various forms of cancer.

The fluorescent proteins are important tools for visualizing and monitoring the internal processes within cells. They have been used in the biomedical applications of monitoring various aspects of cancer, such as primary tumor growth, tumor cell motility and invasion, metastatic seeding, colonization, and angiogenesis [14]. In 2006, Bulina and Chudakov et al. have developed the first genetically encoded red fluorescent protein PS, KillerRed, which opened a new area of fluorescent proteins application [15]. KillerRed was engineered from non-fluorescent and non-phototoxic chromoprotein anm2CP from *Hydrozoa* jellyfish, with the fluorescence excitation and emission maxima at 585 and 610 nm, respectively [16]. Under irradiation with light at the wavelength of 520–590 nm, KillerRed can efficiently produce ROS like superoxide anion radical and H_2_O_2_ [17]. And the ROS-induced photodynamic activity of KillerRed is 1000-fold higher than that of other fluorescent proteins [15]. The unique property of KillerRed could make it used for inactivation of specific proteins by chromophore-assisted light inactivation (CALI) and light-induced cell killing in PDT. Compared to the chemical PSs, the preparation of KillerRed is relatively easier. KillerRed can also be expressed by a target cell, both individually or in fusion with other targeting protein. Therefore, in the present work, we obtained the KillerRed expressed in *Escherichia coli* cells and investigated its photodynamic effects on the cell proliferation and apoptosis of K562 (chronic myelogenous leukemia), NB4 (acute monocytic leukemia), and THP1 (acute monocytic leukemia) cell lines.

## Methods

### Materials

pKillerRed-B prokaryotic expression vector encoding for KillerRed, and rabbit polyclonal antibody against KillerRed were both purchased from Evrogen (Moscow, Russia). *E. coli* BL21(DE3) cells were kindly provided by Prof. Heng Li in the College of Life Science, Northwest University, China. Luria-Bertani (LB) broth, agar, ampicillin, and isopropyl-1-thio-β-D-galactopyranoside (IPTG) were obtained from Solarbio (Beijing, China). Chromatographic column XK16, Q-Sepharose Fast Flow resin were obtained from GE healthcare (Uppsala, Sweden). K562, NB4, and THP1 cell lines were obtained from First Affiliated Hospital of Xi’an Jiaotong University, (Xi’an, China). RPMI medium modified 1640, penicillin, and streptomycin were purchased from Hyclone (Logan City, USA). Fetal bovine serum was obtained from Zhengjiang Tianhang Biotechnology (Hangzhou, China). Hoechst 33342 dye was purchased from Sigma-Aldrich (San Francisco, USA). Cell Counting Kit-8 (CCK-8) was provided by Beijing 4A Biotech (Beijing, China). Pharmingen™ PE Annexin V Apoptosis Detection Kit I was obtained from BD Biosciences (New Jersey, USA). ROS probe 2′,7′-dichlorofluorescein diacetate (H2DCFDA) was purchased from MCE (Shanghai, China). NE-PER Nuclear and Cytoplasmic Extraction Reagents was provided by Thermo scientific (Salem, USA). Rabbit polyclonal antibody against GAPDH and H3 were purchased from Cell Signaling Technology (Danvers, USA) and Abcam (Cambridge, UK), respectively.

### Instruments

Sodium dodecyl sulfate polyacrylamide gel electrophoresis (SDS-PAGE) was conducted on a Junyi electrophoresis system (Beijing, China). Purification of protein was performed on a GE ÄKTA purifier fast protein liquid chromatography (FPLC) (Uppsala, Sweden). An Amicon ultrafiltration cell equipped with a YM-10 cellulose membrane was used for the concentration of KillerRed (Darmstadt, Germany). Electroblotting was conducted on a Bio-Rad Trans-Blot SD Semi-Dry Transfer Cell (Berkeley, USA). The absorption spectra were recorded on a Thermo Fisher 1510 Spectrophotometer (Waltham, USA). Light irradiation experiments were performed under a Ceaulight CEL-HXF300 system (Beijing, China). A wavelength range between 400 and 780 nm was selected by a Ceaulight CEL-UVIRCUT PD-145 optical filter (Beijing, China). Flow cytometry analysis was measured on a Beckman Counter CytoFLEX Flow Cytometer (Suzhou, China). Fluorescent Imaging was recorded on a Carl Zeiss LSM700 confocal laser scanning microscope (CLSM, Oberkochen, Germany).

### Expression of KillerRed

The pKillerRed-B vector was transfected into *E. coli* BL21(DE3) cells by CaCl_2_ method. The colonies containing the vector were selected on LB agar plate supplemented with 25 μg/mL ampicillin, and then inoculated into 50 mL LB broth containing 25 μg/mL ampicillin. After the pre-culture overnight at 37 °C, 10 mL of the culture was transferred into 1 L of LB broth containing 25 μg/mL ampicillin. The cells were grown at 37 °C with shaking to an optical density at 600 nm (OD_600 nm_) of 0.6, then IPTG was added into the culture to a final concentration of 1 mM. The cells were continued to shake at 37 °C for 24 h, and collected by centrifugation at 4000 rpm for 20 min. After discarding the supernatant, bacterial cells were resuspended in 20 mL of Tris-HCl buffer (30 mM, pH 7.6).

### Purification of KillerRed

Bacterial cells in 20 mL of Tris-HCl buffer (30 mM, pH 7.6) were ruptured by ultrasonication (30 mW, 20 min) on ice. The resulting suspension were centrifuged at 9000 rpm for 30 min. The cell debris was discarded and the supernatant was loaded onto an previously equilibrated 20 × 200 mm Q-Sepharose Fast Flow column on GE ÄKTA purifier FPLC system. Bound KillerRed was eluted from the column with a linear gradient of 0–400 mM NaCl in 30 mM Tris-HCl buffer, pH 7.6, at a flow rate of 2 mL/min, for 4 h. Fractions containing KillerRed were identified by 10% SDS-PAGE. And the pure KillerRed was concentrated with the Amicon ultrafiltration cell. KillerRed concentrations were determined using Beer’s law and an extinction coefficient of 45,000 M^− 1^ cm^− 1^ at 280 nm on the spectrophotometer [15].

### Western-blotting analysis

KillerRed was resolved on 10% SDS-PAGE and transferred to polyvinylidene difluoride membrane (PVDF, Bio-Rad, USA). PVDF membrane was washed with tris-buffered saline containing 0.1% Tween 20 (TBST) and blocked with 5% non-fat milk in TBST. The blots were probed with KillerRed polyclonal antibody at 4 °C overnight, then detected by chemiluminescence using Goat anti-Rabbit Secondary Antibody and Westar ECL-Sun substrate (CYANAGEN, Italy).

### Cell culture

The K562 and THP1 cells were obtained from ATCC (CCL-243 and TIB-202, Manassas, VA, USA) and the NB4 cells were obtained from BeNa Culture collection (BNCC337678, Beijing, China). These cell lines were authenticated by the provider, and were tested each month for mycoplasma contamination using the LookOut Mycoplasma PCR Detection Kit (Thermo Scientific, Salem, USA). The K562, NB4, and THP1 cells were cultured in RPMI medium 1640, supplemented with 100 U/mL penicillin, 100 μg/mL streptomycin, and 10% fetal bovine serum in culture dishes at 37 °C in a humidified atmosphere of 5% CO_2_.

### Photodynamic treatment

KillerRed obtained in Tris-HCl buffer was dialyzed three times against 1 L of sterilized phosphate-buffered saline (PBS) at 37 °C for 12 h, and concentrated to 100 μM for further use. For photodynamic treatment experiments, leukemia cells were plated in 96-well plates (Corning, USA) at density of 3 × 10^4^ cells per well and cultured overnight. The cells were centrifuged (1500 rpm for 10 min), washed with sterilized PBS twice, and incubated with sterilized PBS containing different concentrations of KillerRed (0.01–10 μM) at 4 °C for 1 h in the dark. After irradiation with 400–780 nm white light (80 mW cm^− 2^) for 5, 15, and 25 min (total energy dose of 24, 72 J, and 120 cm^− 2^, respectively), PBS was substituted for the RPMI medium 1640, and the cells were incubated for an additional 12 h at 37 °C in 5% CO_2_ atmosphere.

### Cell proliferation assay

Cell proliferation assay was detected using the CCK-8 kit according to the manufacturer’s instructions. After the photodynamic treatment and incubation for 12 h, 10 μL of CCK-8 solution was added to each well, and the plates were incubated at 37 °C for 1 h. The absorbance was measured by a spectrophotometer at the wavelength of 450 nm. Cell survival rate was calculated using the following equation and each assay was performed in quadruplicate.

$$ \mathrm{Cell}\kern0.5em \mathrm{Survival}\kern0.5em \mathrm{Rate}\kern0.5em \left(\%\right)\kern0.5em =\kern0.5em \left(1\hbox{-} \frac{\mathrm{Abs}\kern0.5em \left(\mathrm{no}\kern0.5em \mathrm{treatment}\right)\kern0.5em \hbox{-} \kern0.5em \mathrm{Abs}\kern0.5em \left(\mathrm{PDT}\kern0.5em \mathrm{treatment}\right)}{\mathrm{Abs}\kern0.5em \left(\mathrm{no}\kern0.5em \mathrm{treatment}\right)\kern0.5em \hbox{-} \kern0.5em \mathrm{Abs}\kern0.5em \left(\mathrm{medium}\right)}\right)\kern0.5em \times \kern0.5em 100\% $$

### Cell apoptosis assay

Cell apoptosis assay was detected using the Pharmingen™ PE Annexin V Apoptosis Detection Kit and flow cytometry. Briefly, leukemia cells (1 × 10^6^ cells/mL, 2 mL) were placed in 6-well plates (Corning, USA), incubated with 1 μM of KillerRed in sterilized PBS at 4 °C for 1 h in the dark, and irradiated with 400–780 nm white light for 15 min. After photodynamic treatment and a 12 or 24 h period of incubation, cells were washed twice with ice-cold sterilized PBS, centrifuged at 1500 rpm for 10 min, and resuspended in 1 × PE Annexin V Binding Buffer (0.01 M HEPES/NaOH, 0.14 M NaCl, 2.5 mM CaCl_2_, pH 7.4,) at a concentration of 1 × 10^6^ cells/mL. Afterwards, 100 μL of the cell suspension (containing 1 × 10^5^ cells) was transferred to a culture tube, followed by adding 5 μL of PE Annexin V and 5 μL of 7-Amino-Actinomycin (7-AAD). After a gentle vortex and incubation at 25 °C for 15 min in the dark, 400 μL of Binding Buffer was added. Finally, cell apoptosis was detected by the flow cytometer immediately. All data were analyzed with CytExpert software.

### ROS measurement

To quantitatively measure the formation of ROS, the cell lines (1 × 10^6^ cells/mL, 2 mL) were placed in 6-well plates, incubated with 1 μM of KillerRed in PBS at 4 °C for 1 h in the dark. After that the cells were irradiated with 400–780 nm white light (80 mW cm^− 2^) for 15 min. Then the cells were resuspended in PBS, incubated with 5 μM of H2DCFDA in the dark for 37 °C, which can be easily oxidized to the green fluorescent 2′, 7′-dichlorofluorescein (DCF) by intracellular ROS, and immediately analyzed by flow cytometry. The cell lines treated with 10 mM H_2_O_2_ for 1 h were regarded as the positive control. All data were analyzed with CytExpert software.

### Confocal laser scanning microscopy (CLSM)

Leukemia cells were incubated with 10 μM of KillerRed in sterilized PBS at 4 °C for 4 h in the dark, and irradiated with 400–780 nm white light for 15 min. The non-irradiated and irradiated cells were washed with ice-cold sterilized PBS and fixed in 4% paraformaldehyde solution at 25 °C for 15 min. After that, the cells were permeabilized with 0.2% Triton X-100 for 10 min, stained with 10 μg/ml Hoechst 33342 for 5 min. Finally, the cells were washed with PBS and observed on the CLSM with emission wavelengths of 555 nm.

### Cytoplasmic and nuclear protein extraction assay

NE-PER Nuclear and Cytoplasmic Extraction Reagents was employed to extract cytoplasmic and nuclear protein. K562, NB4 and THP1 cells (1 × 106 cells/mL, 2 mL) were seeded on a 6-well culture dish, and were treated with PBS containing 10 μM of KillerRed for 1 h at 4 °C. After irradiation with 400–780 nm white light (80 mW cm-2) for 15 min, the cytoplasmic and nuclear proteins were extracted according to the manufacturer’s instructions. Next, western blotting assay was performed as previously described.

### Statistical analysis

All experiments were repeated at least three times. Data were expressed as mean ± standard deviation (SD). Statistical analysis was performed using the ANOVA (Bonferroni’s post-test) analysis by GraphPad prism5 software to evaluate the significance of the difference between groups, and described as: * *p* < 0.05, ** *p* < 0.01, and *** *p* < 0.001.

## Results

### Expression and purification of KillerRed

**Fig. 1:**
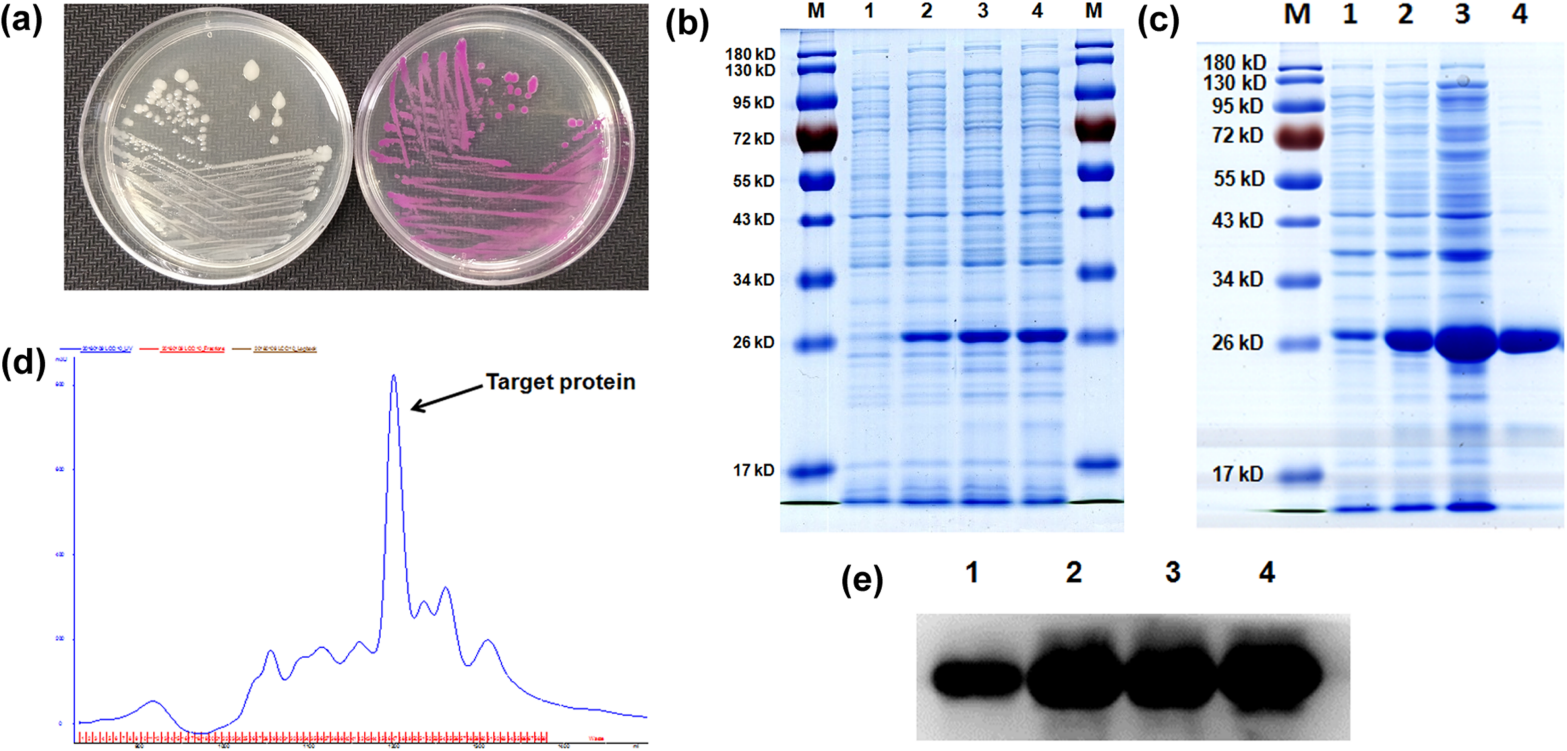
Expression and purification of KillerRed. **a**: Images of *E. coli* BL21(DE3) (left) and *E. coli* BL21(DE3) containing pKillerRed-B vector (right); **b**: SDS-PAGE gel of KillerRed expression. Lane M: molecular marker; lane 1: *E. coli* BL21(DE3) cells; lane 2: pKillerRed-B contained *E. coli* BL21(DE3) cells after IPTG induction for 4 h; lane 3: cells after IPTG induction for 10 h; lane 4: cells after IPTG induction for 24 h. **c**: SDS-PAGE gel of KillerRed purification. Lane M: molecular marker; lane 1: pKillerRed-B contained *E. coli* BL21(DE3) cells without IPTG induction; lane 2: cells after IPTG induction for 24 h; lane 3: supernatant of ultrasonication-treated cells; lane 4: purified KillerRed eluted from Q-Sepahrose Fast Flow column. **d**: FPLC elution profile of KillerRed. **e**: Western-blotting analysis of KillerRed. Lane 1–4: the same as (**c**)

The pKillerRed-B vector was transfected into *E. coli* BL21(DE3) cells. Bacterial cells containing pKillerRed-B vector were selected on LB agar plates supplemented with 25 μg/mL ampicillin. After 24 h incubation at 37 °C, the developed colonies showed red color, indicating that the bacteria can express KillerRed efficiently (as shown in Fig. 1a). The SDS-PAGE analysis indicated that a band around 26 kD emerged in bacterial cells containing the vector. The over-expression of KillerRed was induced by adding 1 mM of IPTG, and the band around 26 kD increased with the incubation time (Fig. 1b). After ultrasonication of bacterial cells and centrifugation, we found that almost all target protein was existing in the supernatant, suggesting that KillerRed expressed is soluble (Fig. 1c). Due to the isoelectric point (pI) of KillerRed of 5.24, a 20 × 200 mm Q-Sepharose Fast Flow column on GE ÄKTA purifier FPLC was chosen for purification of KillerRed. The column was eluted with a linear gradient of 0–400 mM NaCl in 30 mM Tris-HCl buffer, at a flow rate of 2 mL/min (Fig. 1d), for 4 h. With this method, the yield of KillerRed was about 37 mg per liter of bacterial cells. The purified KillerRed was analyzed by SDS-PAGE (Fig. 1c) and further confirmed by western-blotting (Fig. 1e).

### Photodynamic treatment on cell survival

**Fig. 2:**
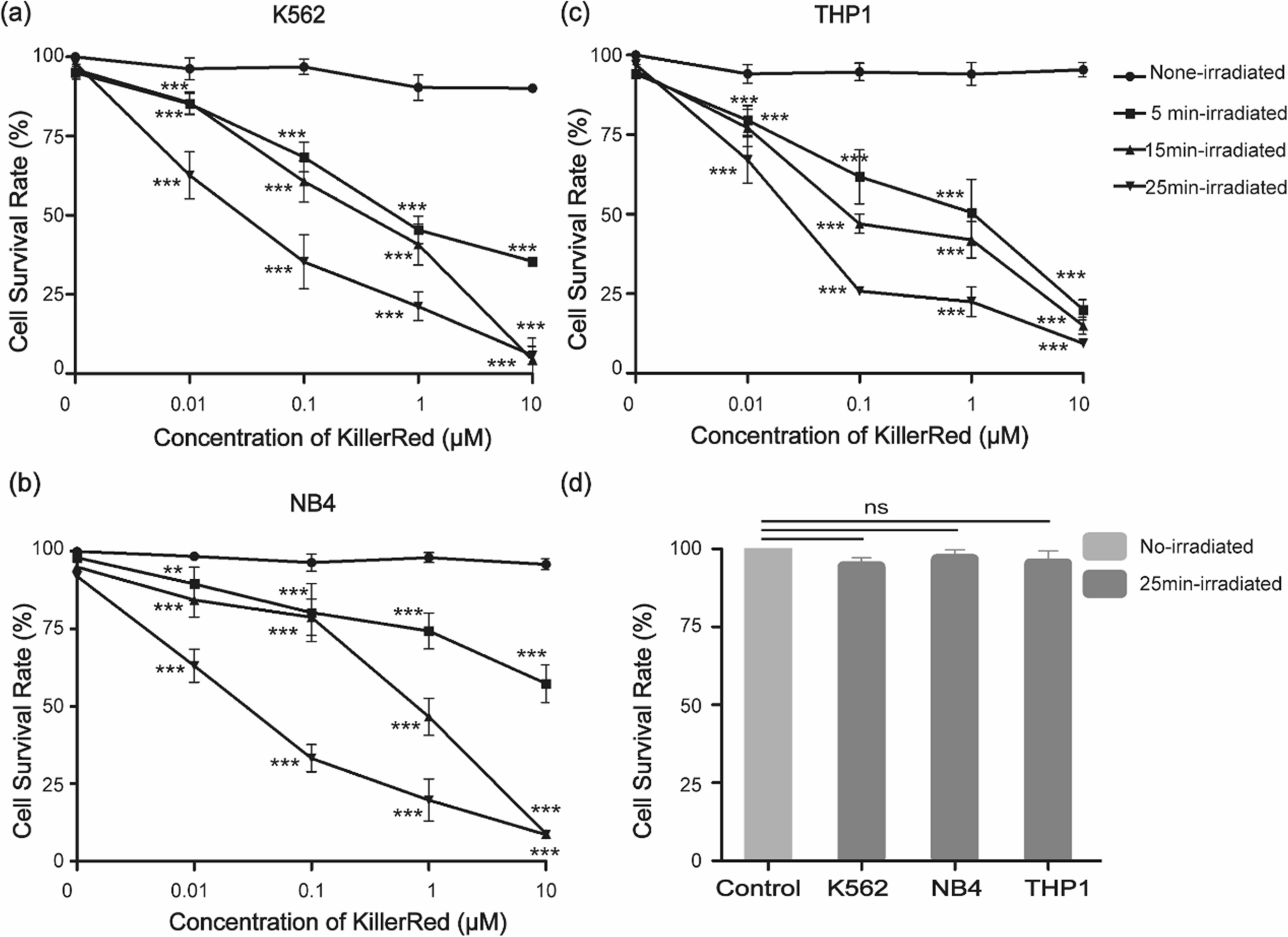
KillerRed-mediated PDT effects on the cell proliferation (CCK-8 assay). **a-c**: Cells were treated with KillerRed (0.01–10 μM) and irradiated with 400–780 nm light for 5, 15, and 25 min (24, 72, 120 J cm^− 2^). **d**: Cells were irradiated with light for 25 min alone (120 J cm^− 2^). Values are means ± standard deviation of three replicates (*t*-test comparing to the control cells without treatment)

A CCK-8-based colorimetric cell proliferation assay was used to assess the photodynamic cytotoxicity of KillerRed on leukemia cells. Increasing concentrations of KillerRed were added to the leukemia cells, and after a 1 h incubation at 4 °C and irradiation with white light for 5, 15, and 25 min, proliferation of these cells was determined compared with untreated cells. As shown in Fig. 2, for the cells treated with KillerRed but without light irradiation, a significant decrease in cell survival rate was not observed, indicating that KillerRed did not exhibit obvious dark toxicity for the three leukemia cells at the tested concentrations. Furthermore, direct exposure of these cells to light in the absence of KillerRed produced no significant cytotoxic effect. In contrast, the irradiated groups showed reduced cell survival rates with increasing concentrations of KillerRed and light doses. After incubation with 10 μM of KillerRed and irradiation with 120 J cm^− 2^ light, the survival rates of K562, NB4, and THP1 cells decreased to 5.80, 8.57 and 9.34%**,** respectively, indicating that over 90% of leukemia cells were killed.

### Photodynamic treatment on cell apoptosis

**Fig. 3:**
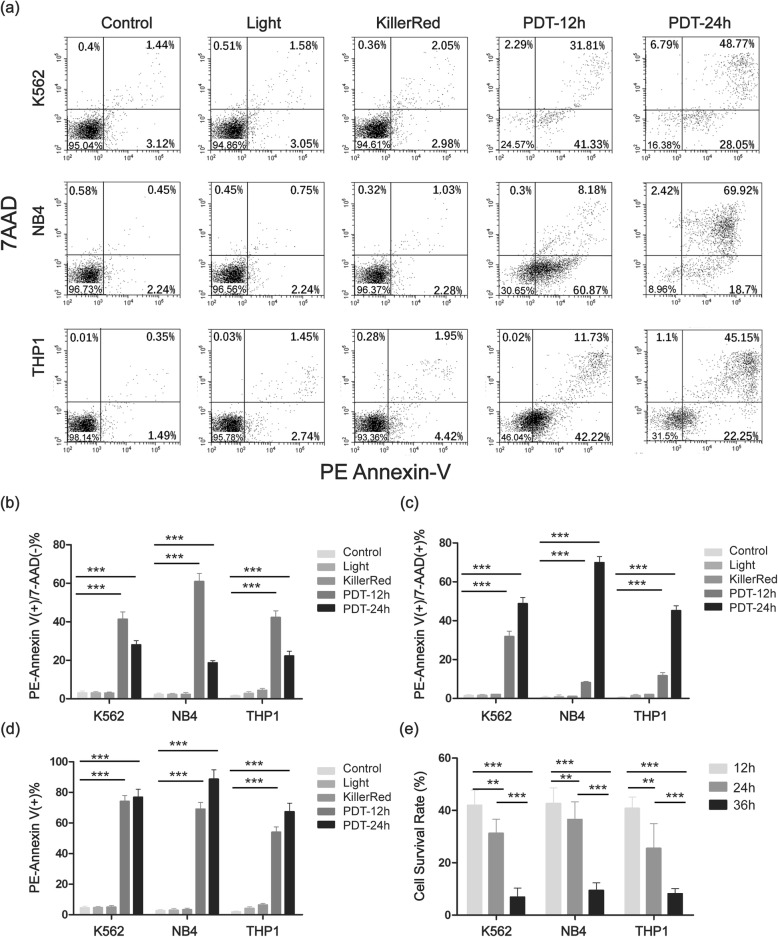
KillerRed-mediated PDT effects on the cell apoptosis (PE Annexin V/7-ADD staining and flow cytometry assay). Data are means±SD of three independent experiments (** *p* < 0.05 and ***p < 0.01 versus control)

In an effort to determine whether KillerRed-mediated PDT could induce apoptosis of the leukemia cells, PE Annexin-V/7-AAD staining and flow cytometry analysis were performed. As plotted in Fig. 3, the apoptosis rates of cells treated with KillerRed alone and cells irradiated with white light alone did not show apparent difference with that of cells without any treatment. However, after incubation with 1 μM of KillerRed and irradiation with 72 J cm^− 2^ white light, the apoptotic cell population obviously increased compared with the control group. To be specific, the apoptotic cells of K562 increased from 4.6 to 73.1% (with 41.3% early apoptotic cells and 31.8% late apoptotic cells) after 12 h incubation, and increased to 76.82% (with 28.05% early apoptotic cells and 48.77% late apoptotic cells) after 24 h incubation. The apoptotic cells of NB4 increased from 2.70 to 69.1% (with 60.9% early apoptotic cells and 8.2% late apoptotic cells) after 12 h incubation, and increased to 88.62% (with 18.7% early apoptotic cells and 69.92% late apoptotic cells). The apoptotic cells of THP1 increased from 1.8 to 54.0% (with 42.2% early apoptotic cells and 11.7% late apoptotic cells) after 12 h incubation, and increased to 67.4% (with 22.25% early apoptotic cells and 45.15% late apoptotic cells). After incubation for 36 h, over 90% of the K562, NB4, and THP1 cells were dead and could not be analyzed by flow cytometry.

### The generation of intracellular ROS

**Fig. 4:**
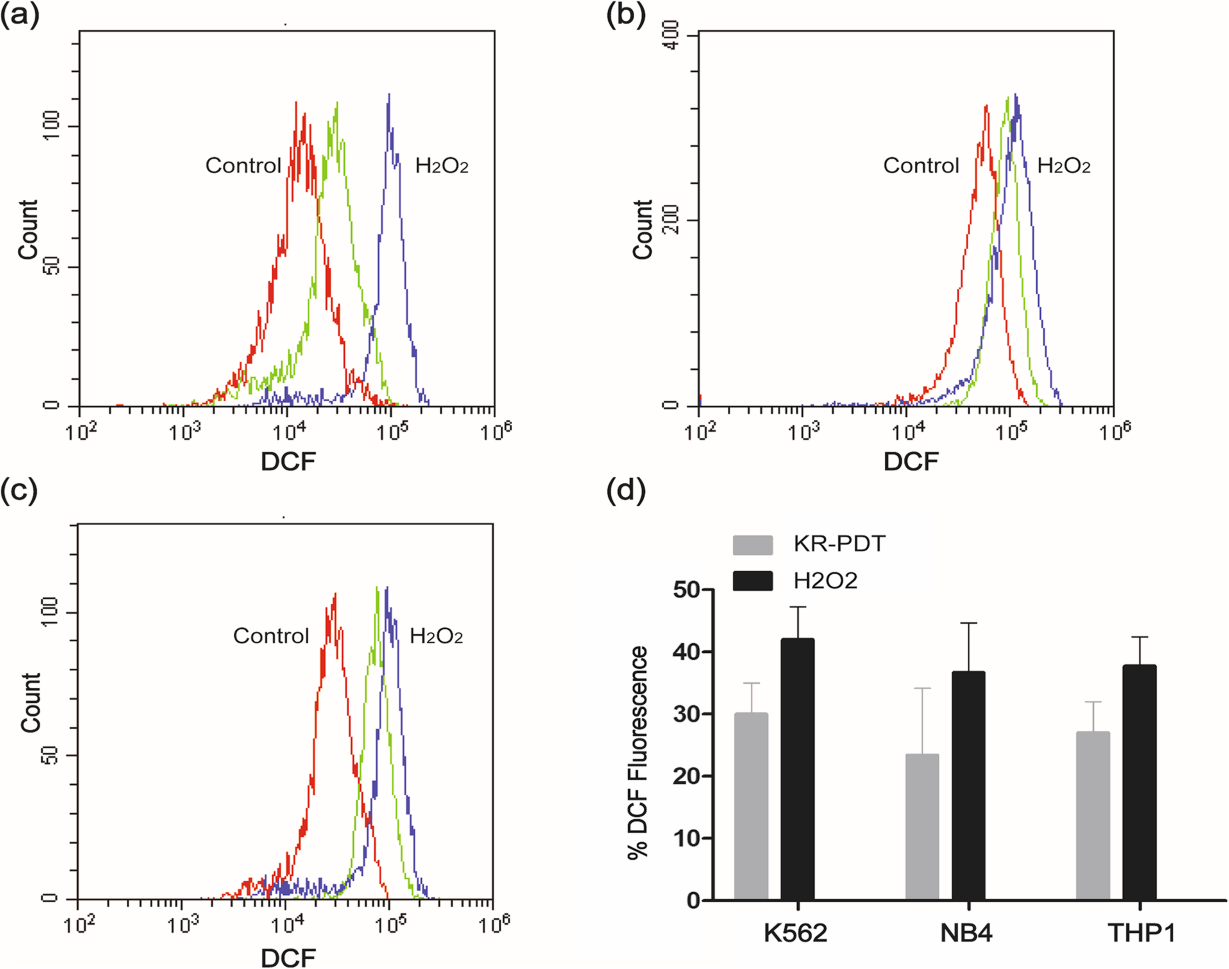
KillerRed-mediated PDT induced intracellular ROS in K562 (**a**), NB4 (**b**), and THP1 (**c**) cell line. The green curve represents cells treated with KillerRed-mediated PDT. The red curve represents cells treated with KillerRed alone. H_2_O_2_ was used as a positive control (blue curve). **d** Results are expressed as means ±SD from three independent experiments

We detected the fluorescent DCF by flow cytometry to monitor the generation of intracellular ROS. The results showed that exposure of cells to KillerRed-mediated PDT resulted in significantly increased ROS production, as compared to the cells treated with KillerRed alone (Fig. 4). The level of ROS increased 30% in K562 cell line, 23.4% in NB4 cell line, and 27% in THP1 cell line, indicating that KillerRed after light irradiation could cause significant accumulation of ROS in these cells.

### The intracellular accumulation of KillerRed

**Fig. 5:**
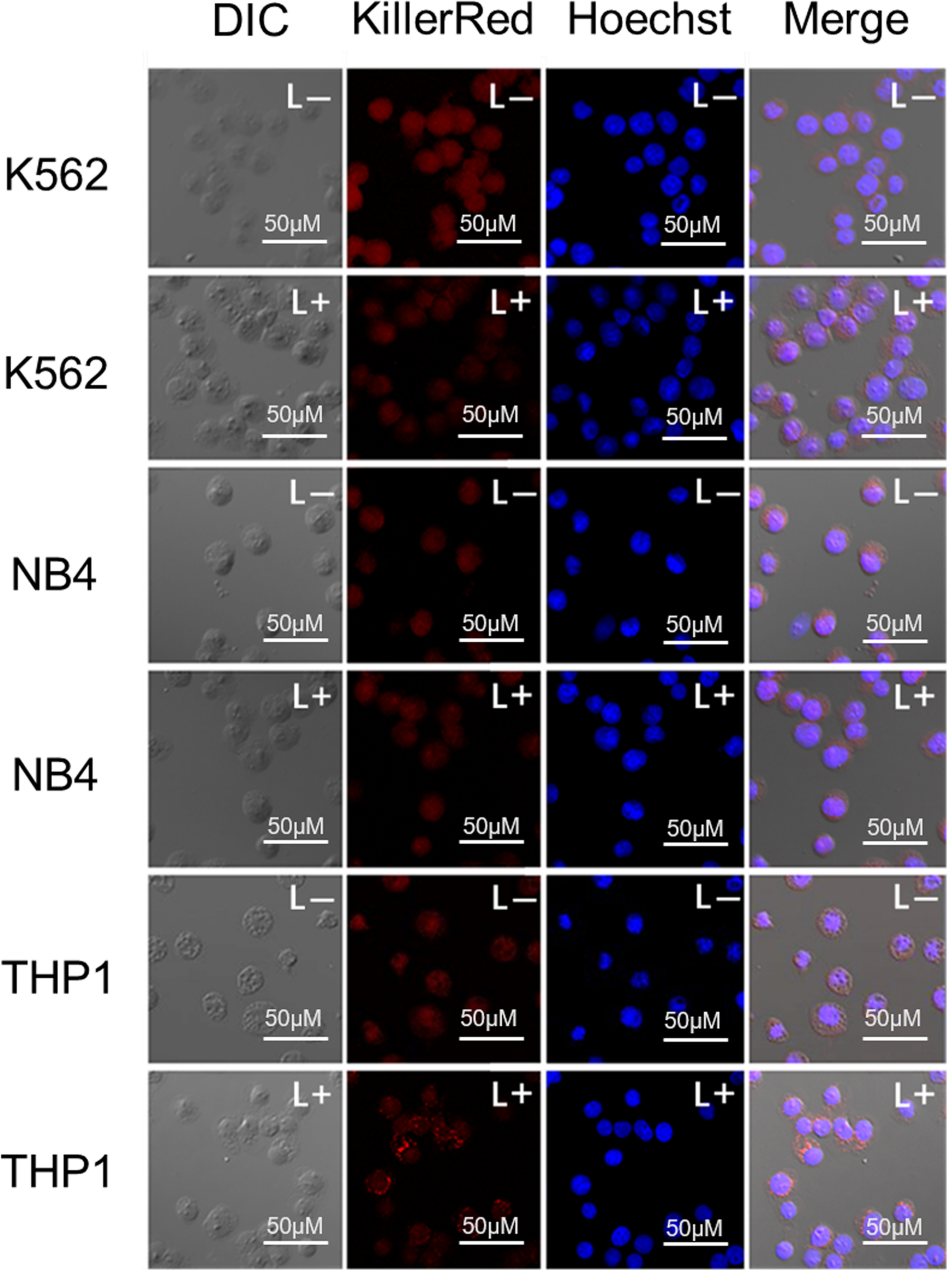
CLSM images of K562, NB4, and THP1 cell lines incubated with 10 μM of KillerRed at 4 °C for 4 h in the dark. L-: without light irradiation; L+: irradiated with 400–780 nm light for 15 min (72 J cm^− 2^). Hoechst 33342 was used for nuclei staining

CLSM was used to explore more information on the intracellular distribution of KillerRed in K562, NB4 and THP1 cells. The cells were incubated with KillerRed at 4 °C for 4 h, and the nuclei of cells was counterstained with Hoechst 33342 (blue fluorescence). As shown in Fig. 5, the red fluorescence of KillerRed could be detected in the entire cells of K562, NB4 and THP1, before and after white light irradiation, suggesting that KillerRed could enter into the cytoplasm and nuclei of cells with a non-specific mode. The nuclei was clearly differentiated as round blue fluorescence, which showed no obvious difference in non-irradiated and irradiated cells, indicating that the nuclear envelope kept intact during the photodynamic treatment. In addition, the condensation of red fluorescence observed in individual cells (especially THP1 cells) after light irradiation suggested that KillerRed-mediated photodynamic treatment could cause damage to the cytoplasm of leukemia cells.

**Fig. 6:**
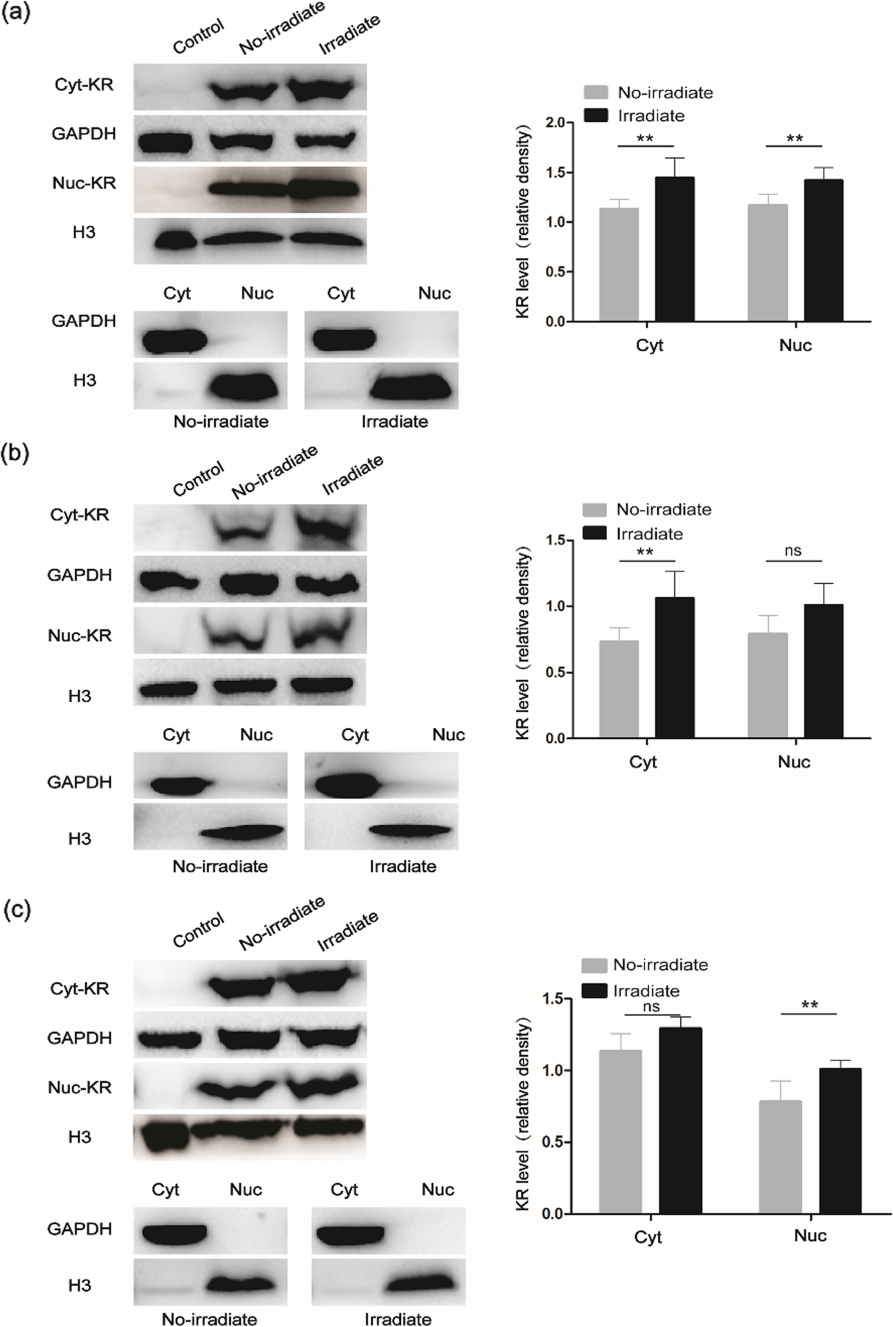
KillerRed level in cytosol and nuclei of K562 (**a**), NB4 (**b**), and THP1 (**c**) cells investigated by Western blots. The data were quantified by ImageJ software and the percentage of KillerRed level related to GAPDH (cytosol) or H3 (nuclei) were presented as means ± SD (*n* = 3). **denotes *P* < 0.05. ns denotes no significant difference

In consistent with the results of CLSM, KillerRed could be detected both in the cytosol and nuclei for the three cell lines by western blotting analysis (Fig. 6). Furthermore, although some differences were not statistically significant, KillerRed level increased in cytosol and nuclei after light irradiation.

## Discussion

So far, the main treatments for leukemia have certain defects. Higher doses of radiotherapy have diverse side effects during treatment, even in the years following treatment and it may not completely destroy the cancer cells. Chemotherapy can cause a range of side-effects and be impaired by drug-resistance. Transplantation of allogenous hematopoietic stem cells have strong risks for recipients and donors [18]. PDT is a minimally invasive and complementary therapeutic procedure for cancers and other diseases. It has been abundantly researched and already used in clinical trials for treating various kinds of malignancy, including skin tumors, head and neck tumors, digestive system tumors, intraperitoneal malignancies, urinary system tumors, non-small cell lung cancer, and brain tumors [19]. Clinical studies revealed that PDT can prolong survival in patients with inoperable cancers and significantly improve quality of life. Advantages like minimal normal tissue toxicity, negligible systemic effects, greatly reduced long-term morbidity, and lack of intrinsic or acquired resistance mechanisms make this treatment as a valuable therapeutic option for combination treatments [19]. However, relative less attention has been focused on employing this technique for treating leukemia. Few previous studies indicated that chronic myelogenous and acute monocytic leukemia cells could be effectively killed under light irradiation by several classical PSs such as 5-aminolevulinic acid (5-ALA), zinc phthalocyanine (ZnPc), and hypericin [20–22].

In an effort to demonstrate the photodynamic effects of KillerRed on leukemia cells, the present study performed KillerRed-mediated PDT experiments using three kinds of leukemia cell lines. The results showed that both KillerRed and light irradiation alone caused no significant cytotoxic effect on the three cell lines. KillerRed combined with light irradiation inactivated these cell lines in a concentration and light dose-dependent manner. After incubation with 10 μM of KillerRed and irradiation with 120 J cm^− 2^ light, over 90% of leukemia cells were killed. K562 cell line is prevalently employed in the study of chronic myelogenous leukemia. Pluskalova et al. found that 5-ALA mediated PDT could significantly reduce the growth of K562 cells by 2.7-fold compared to the untreated cells. The expression of the oncogenic BCR-ABL1 kinase was suppressed and the cytoskeleton organization of K562 cells was affected during the PDT treatment [20]. In another study by Xu et al., PDT based on hypericin, a naphthodianthrone structure PS isolated from *Hypericum* species, could induce apoptosis in K562 human leukemia cells through JNK pathway modulation. The results indicated that 0.8 μg/mL (1.58 μM, the MW of hypericin is 504.44) under 72 J cm^− 2^ of light could reduce the cell survival rate of K562 cells to about 30% [22]. In the present study, after incubation with 1 μM of KillerRed and irradiation with the same dose of light, the survival rates of K562 cells decreased to 40.8%, suggesting that the photodynamic efficacy of KillerRed was a little lower than that of hypericin. We have assessed the KillerRed-mediated PDT on the acute monocytic leukemia NB4 cells, and the photodynamic efficacy was comparable to that of K562 cells, which was different with the 5-ALA-mediated PDT reported by Li et al., who demonstrated that it was effective in NB4 cells, which accumulated high levels of protoporphyrin IX (PpIX), but not effective in K562 cells, which accumulated relatively low levels of PpIX. They also found that multidrug-resistant (MDR) NB4 cells was also sensitive to 5-ALA-mediated PDT and the susceptibility was not affected or impaired in any way by their resistance to anticancer drugs [23]. In addition, KillerRed-mediated PDT was also effective in killing acute monocytic leukemia THP1 cells, and 10 μM of KillerRed combined with 72 J cm^− 2^ light could yield 85% decrease in cell survival. To the best of our knowledge, this is the first time of using this cell line in the PDT research.

We used PE Annexin-V/7-AAD staining and flow cytometry to investigate the photodynamic treatment on cell apoptosis. In apoptotic cells, PE Annexin V staining can identify apoptosis at an earlier stage than other assays based on nuclear changes such as DNA fragmentation. 7-AAD can enter into the dead or membrane damaged cells but is excluded by viable cells with intact membranes. Thus, PE Annexin V combined with 7-AAD can identify early and late apoptotic cells. In this study, K562, NB4, and THP1 cells were incubated with an intermediated concentration of KillerRed (1 μM) and irradiated with 72 J cm^− 2^ white light. After PDT treatment, the majority of K562, NB4, and THP1 apoptotic cells were in early stage, suggesting that the membrane phospholipid phosphatidylserine of these cells was affected during the PDT treatment. Minority of apoptotic cells were in late apoptosis stage indicating that the membranes integrity of these cells had been destroyed. The results of the PE Annexin V/7-AAD assay demonstrated that KillerRed-mediated PDT predominantly triggered early apoptotic response in the three leukemia cells, which was similar to sinoporphyrin-mediated PDT on breast cancer MDA-MB-231 cells reported by Wu and co-workers [24].

We used H2DCFDA as a probe in combination with flow cytometry to detect the generation of intracellure ROS after PDT treatment. The results showed that ROS level significantly increased in PDT treated cells compared to the cells treated with KillerRed alone. In the present study, cell survive reduction, apoptosis and necrosis, and cytoplasm damage were induced by ROS generated from KillerRed after light irradiation. ROS has been demonstrated to result in DNA damage, cellular apoptosis related with mitochondria, etc. [25, 26]. However, the mechanism of KillerRed-mediated PDT inactivating leukemia cells was still unclear and need further investigation.

Abdulrehman et al. reported that m-THPC, a powerful second generation PS, was mainly distributed within the endoplasmic reticulum and lysosome of SW480 colon cancer cells, and within the lysosome and mitochondria of SW620 metastatic colon cancer cells [27]. Wu et al. found that palmatine, a naturally occurring PS isolated from traditional Chinese medicine rhizomes of *Fibrarurea Tinctoria Lour*, mainly located in mitochondria and endoplasmic reticulum of MCF-7 breast cancer cells [28]. Du et al. reported that chlorophyllin f showed affinity for mitochondria and lysosome in 5637 and T24 bladder cancer cells [29]. From above reported studies we found that mitochondria, lysosome, and endoplasmic reticulum, but not nuclei, were the main targets for these chemical PSs, which could induce damages on these cell organelles after light irradiation. However, in this study, we observed that protein PS KillerRed was distributed not only within the cytoplasm but also within the nuclei of leukemia cells, which was confirmed by CLSM and western-blotting. Furthermore, KillerRed level was improved after light irradiation, suggesting that an increased cell permeability and uptake of KillerRed after PDT treatment.

After light irradiation, the CLSM images showed that the nuclear envelope kept intact and the KillerRed fluorescence condensed in individual cells. From these results we speculated that KillerRed-mediated PDT could directly produce strong damages to the cytoplasm in the irradiation process. In previous study by Xu et al., post hypericin-mediated PDT treatment, the K562 cells after 4 h incubation at 37 °C presented typical apoptotic cell characteristics, including a concentrated cytoplasm, bubble-like protrusions on the cell surface, the formation of apoptotic bodies. Although chromatin condensation, agglomeration at the central nuclear area could be observed by transmission electron microscope, the nuclear membrane was not destroyed. However, cells after 16 h incubation at 37 °C showed loss of intracellular detail and destroyed nuclear membrane [22].

Autologous hematopoietic stem cell transplantation is a promising approach for leukemia therapy. Compared to allogenous hematopoietic stem cell transplantation, it possesses several advantages such as easy transplantation of grafts, little restriction of age, little need for HLA-matched donor, the absence of graft-versus-host disease, and a small percentage of life-threatening infections [21]. However, autologous hematopoietic stem cell transplantation still has its own limitation of high relapse rate which was ascribed to the risk of reinfusing residual leukemia cells at the time of harvest. PDT-based ex vivo purging is an emerging technique for transplantation of autologous hematopoietic stem cells in the treatment of leukemia. Huang et al. investigated the purging efficacy of ZnPc-based PDT on leukemia bone marrow, and found that leukemia cells exhibited higher susceptibility to ZnPc-mediated PDT than normal granulocyte/macrophage progenitors, as well as eliminate K562 cells from the mixture of K562 and normal bone marrow mononuclear cells [21]. Unlike chemical PSs, KillerRed can be targeted to specific cell compartments using well-known localization signals due to the nature of a genetically encoded PS. In several previous studies, different vectors were constructed and transfected into tumor cells for localized expression of KillerRed in different cell organelles such as mitochondria, plasma membrane, lysosome, and chromatin [16, 30–32].

One limitation of this study is that we only investigated the photodynamic effect of KillerRed on three leukemia cell lines, but lack of comparison with normal bone marrow haematopoietic cells. The selectivity of KillerRed on leukemia cells over normal bone marrow haematopoietic cells is still unclear. In order to enhance it, one strategy is employing targeting moieties. For instance, Serebrovskaya et al. reported a fully genetically encoded immuno-PS, consisting of a specific anti-p185^HER-2-ECD^ antibody fragment 4D5scFv fused with KillerRed for targeted PDT of cancer cells. This fusion protein can efficiently kill p-185^ER-2-ECD^-expressing cancer cells upon light irradiation with fine targeting properties [33]. In this way, the selectivity and killing efficacy of KillerRed-mediated PDT could be further enhanced. The present study demonstrated that KillerRed alone combined with light irradiation could effectively eliminate chronic myelogenous, acute monocytic, and acute monocytic leukemia cell lines. Our future work will be focused on investigating the selectivity of KillerRed on leukemia cells and normal bone marrow haematopoietic cells, as well as developing novel KillerRed-based fusion PS for targeted photodynamic treatment of leukemia cells, which might be a promising modality, individually or complementally, for leukemia treatments.

## Conclusions

In summary, this work investigated the KillerRed-mediated PDT effect on K562, NB4, and THP1 leukemia cells in vitro. KillerRed exhibited no obvious dark toxicity, and eliminated these cells upon light irradiation in a concentration and light dose-dependent manner. KillerRed-mediated PDT could predominantly trigger early apoptotic response in these cells. KillerRed-mediated PDT could cause the accumulation of ROS in the three cell lines. The CLSM indicated that KillerRed was distributed not only within the cytoplasm but also within the nuclei of leukemia cells, and mainly caused damages to the cytoplasm during the PDT treatment. The western blotting confirmed that KillerRed was distributed both in the cytosol and nuclei, and the KillerRed level was improved after irradiation. These findings showed that KillerRed is a promising PS in photodynamic treatment of leukemia cells, and may provide new insights for KillerRed application in PDT. Further in vivo experiments are needed to confirm that KillerRed can meet all requirements for PDT treatment of leukemia in clinical practice.

## Data Availability

Majority of data generated in this study are included in this publication. Remaining raw data are available from the corresponding author on reasonable request.

## Abbreviations

1O2: Singlet oxygen
5-ALA: 5-aminolevulinic acid
7-AAD: 7-Amino-Actinomycin
CALI: Chromophore-assisted light inactivation
CCK-8: Cell Counting Kit-8
CLSM: Confocal laser scanning microscopy
DCF: 2′,7′-dichlorofluorescein
FPLC: Fast protein liquid chromatography
GVHD: Graft-versus-host disease
H2DCFDA: 2′,7′-dichlorofluorescein diacetate
IPTG: Isopropyl-1-thio-β-D-galactopyranoside
LB: Luria-Bertani
MDR: Multidrug-resistant
NCCR: National Central Cancer Registry of China
PBS: Phosphate- buffered saline
PDT: Photodynamic therapy
pI: isoelectric point
PpIX: protoporphyrin IX
PS: Photosensitizer
PVDF: Polyvinylidene difluoride membrane
ROS: Reactive oxygen species
SD: Standard deviation
SDS-PAGE: Sodium dodecyl sulfate polyacrylamide gel electrophoresis
ZnPc: Zinc phthalocyanine
